# Supplementary material for: Hot spot prediction in protein-protein interactions by an ensemble system
Source: BMC Syst Biol. 2018 Dec 31;12(Suppl 9):132. doi: 10.1186/s12918-018-0665-8 (PMC6311905; doi:10.1186/s12918-018-0665-8)
Supplement: Supplementary file 2 — All relevant MATLAB code. A simple Matlab implement of our predictor. (ZIP 19,742 kb) [file 12918_2018_665_MOESM2_ESM.zip › Additional file 2/Read ASA/ASA website/SKEMPI/1DVFA.htm]

 NetSurfP - Protein Surface Accessibility and Secondary Structure Predictions

  

|  |  |  |
| --- | --- | --- |
|  |  | NetSurfP - Protein Surface Accessibility and Secondary Structure Predictions  Technical University of Denmark |

  


---

```
# For publication of results, please cite:
# A generic method for assignment of reliability scores applied to solvent accessibility predictions.
# Bent Petersen, Thomas Nordahl Petersen, Pernille Andersen, Morten Nielsen and Claus Lundegaard
# BMC Structural Biology 2009, 9:51 doi:10.1186/1472-6807-9-51
#
# Column 1: Class assignment - B for buried or E for Exposed - Threshold: 25% exposure, but not based on RSA
# Column 2: Amino acid
# Column 3: Sequence name
# Column 4: Amino acid number
# Column 5: Relative Surface Accessibility - RSA
# Column 6: Absolute Surface Accessibility
# Column 7: Z-fit score for RSA prediction
# Column 8: Probability for Alpha-Helix
# Column 9: Probability for Beta-strand
# Column 10: Probability for Coil
E D  1DVFA                  1    0.806 116.159   0.503   0.003   0.003   0.994
B I  1DVFA                  2    0.285  52.706  -0.625   0.004   0.514   0.481
E V  1DVFA                  3    0.459  70.548   0.921   0.002   0.816   0.182
B L  1DVFA                  4    0.154  28.124   0.516   0.002   0.816   0.182
E T  1DVFA                  5    0.437  60.640   0.331   0.003   0.718   0.279
B Q  1DVFA                  6    0.151  26.879   0.483   0.004   0.420   0.576
E S  1DVFA                  7    0.411  48.134   0.132   0.004   0.085   0.910
E P  1DVFA                  8    0.449  63.685  -0.867   0.018   0.019   0.964
E A  1DVFA                  9    0.519  57.183  -1.230   0.053   0.043   0.903
E S  1DVFA                 10    0.358  41.934  -1.114   0.066   0.296   0.638
B L  1DVFA                 11    0.220  40.282  -0.099   0.023   0.655   0.322
E S  1DVFA                 12    0.407  47.724  -0.637   0.021   0.756   0.223
B A  1DVFA                 13    0.251  27.649   0.015   0.023   0.655   0.322
E S  1DVFA                 14    0.399  46.716   0.296   0.022   0.359   0.619
E V  1DVFA                 15    0.509  78.264   0.051   0.004   0.085   0.910
E G  1DVFA                 16    0.646  50.817  -1.310   0.005   0.045   0.951
E E  1DVFA                 17    0.374  65.373   0.318   0.004   0.138   0.858
E T  1DVFA                 18    0.549  76.119   1.110   0.003   0.718   0.279
B V  1DVFA                 19    0.076  11.697  -0.004   0.001   0.959   0.040
E T  1DVFA                 20    0.406  56.312   1.287   0.001   0.959   0.040
B I  1DVFA                 21    0.051   9.509   0.315   0.001   0.959   0.040
E T  1DVFA                 22    0.370  51.347   0.598   0.001   0.900   0.099
B C  1DVFA                 23    0.027   3.847   0.248   0.002   0.816   0.182
E R  1DVFA                 24    0.434  99.478   1.169   0.002   0.816   0.182
B A  1DVFA                 25    0.271  29.820  -1.264   0.004   0.616   0.381
E S  1DVFA                 26    0.360  42.251  -1.434   0.004   0.420   0.576
E G  1DVFA                 27    0.524  41.239  -2.478   0.004   0.085   0.910
E N  1DVFA                 28    0.447  65.382  -2.477   0.018   0.047   0.935
E I  1DVFA                 29    0.416  77.015  -1.922   0.018   0.088   0.893
E H  1DVFA                 30    0.320  58.208  -1.241   0.020   0.205   0.775
B N  1DVFA                 31    0.245  35.868  -0.902   0.022   0.359   0.619
B Y  1DVFA                 32    0.095  20.344   0.492   0.021   0.756   0.223
B L  1DVFA                 33    0.031   5.676  -0.004   0.018   0.846   0.136
B A  1DVFA                 34    0.072   7.978   0.233   0.018   0.846   0.136
B W  1DVFA                 35    0.033   7.936   0.073   0.021   0.756   0.223
B Y  1DVFA                 36    0.046   9.766   0.319   0.018   0.846   0.136
B Q  1DVFA                 37    0.151  26.933   0.465   0.023   0.655   0.322
B Q  1DVFA                 38    0.094  16.788   0.224   0.022   0.359   0.619
E K  1DVFA                 39    0.405  83.226   1.443   0.005   0.045   0.951
E Q  1DVFA                 40    0.393  70.136  -0.072   0.005   0.015   0.979
E G  1DVFA                 41    0.766  60.284  -1.877   0.003   0.003   0.994
E K  1DVFA                 42    0.451  92.750  -0.156   0.018   0.019   0.964
E S  1DVFA                 43    0.344  40.364  -0.240   0.018   0.047   0.935
B P  1DVFA                 44    0.105  14.843   0.239   0.004   0.197   0.799
E Q  1DVFA                 45    0.280  50.079   0.540   0.003   0.718   0.279
B L  1DVFA                 46    0.089  16.387   0.501   0.001   0.900   0.099
B L  1DVFA                 47    0.028   5.054   0.709   0.001   0.900   0.099
B V  1DVFA                 48    0.019   2.997   0.684   0.001   0.959   0.040
B Y  1DVFA                 49    0.090  19.148   0.104   0.001   0.959   0.040
B Y  1DVFA                 50    0.096  20.472  -0.178   0.001   0.900   0.099
B T  1DVFA                 51    0.149  20.722  -0.157   0.004   0.616   0.381
B T  1DVFA                 52    0.194  26.838  -1.066   0.005   0.262   0.733
E T  1DVFA                 53    0.560  77.630  -1.299   0.018   0.088   0.893
E L  1DVFA                 54    0.434  79.410  -0.077   0.018   0.047   0.935
E A  1DVFA                 55    0.574  63.299  -1.734   0.018   0.047   0.935
E D  1DVFA                 56    0.705 101.663  -1.354   0.018   0.047   0.935
E G  1DVFA                 57    0.581  45.709  -1.323   0.018   0.047   0.935
E V  1DVFA                 58    0.301  46.294   0.096   0.005   0.045   0.951
E P  1DVFA                 59    0.596  84.572  -0.520   0.005   0.045   0.951
E S  1DVFA                 60    0.407  47.689  -1.573   0.004   0.138   0.858
B R  1DVFA                 61    0.265  60.799  -0.203   0.004   0.616   0.381
B F  1DVFA                 62    0.054  10.798  -0.575   0.002   0.816   0.182
B S  1DVFA                 63    0.203  23.745  -0.271   0.001   0.900   0.099
B G  1DVFA                 64    0.131  10.278  -2.593   0.001   0.900   0.099
E S  1DVFA                 65    0.375  43.985   0.277   0.002   0.816   0.182
E G  1DVFA                 66    0.382  30.040  -0.489   0.004   0.514   0.481
E S  1DVFA                 67    0.619  72.523  -1.261   0.004   0.085   0.910
E G  1DVFA                 68    0.710  55.916  -0.888   0.005   0.015   0.979
E T  1DVFA                 69    0.410  56.867  -0.275   0.004   0.197   0.799
E Q  1DVFA                 70    0.386  68.940   0.643   0.002   0.816   0.182
B Y  1DVFA                 71    0.071  15.109  -0.116   0.001   0.900   0.099
B S  1DVFA                 72    0.242  28.327   0.964   0.001   0.959   0.040
B L  1DVFA                 73    0.031   5.676   0.386   0.001   0.959   0.040
E K  1DVFA                 74    0.293  60.188   0.996   0.001   0.959   0.040
B I  1DVFA                 75    0.074  13.635   0.261   0.002   0.816   0.182
E N  1DVFA                 76    0.445  65.075   0.466   0.004   0.514   0.481
E S  1DVFA                 77    0.539  63.147  -0.314   0.004   0.197   0.799
B L  1DVFA                 78    0.154  28.124   0.164   0.004   0.085   0.910
E Q  1DVFA                 79    0.466  83.263   0.682   0.004   0.085   0.910
E P  1DVFA                 80    0.451  63.969  -0.526   0.018   0.088   0.893
E E  1DVFA                 81    0.552  96.365  -0.819   0.053   0.043   0.903
B D  1DVFA                 82    0.209  30.189  -0.431   0.052   0.084   0.864
B F  1DVFA                 83    0.255  51.259   0.167   0.069   0.386   0.545
B G  1DVFA                 84    0.051   3.974  -0.013   0.021   0.756   0.223
B S  1DVFA                 85    0.180  21.096   0.843   0.018   0.846   0.136
B Y  1DVFA                 86    0.040   8.655   1.061   0.018   0.846   0.136
B Y  1DVFA                 87    0.066  14.168   0.921   0.021   0.756   0.223
B C  1DVFA                 88    0.019   2.696   1.230   0.021   0.756   0.223
B Q  1DVFA                 89    0.043   7.626  -0.226   0.023   0.655   0.322
B H  1DVFA                 90    0.107  19.445  -0.010   0.022   0.552   0.426
B F  1DVFA                 91    0.247  49.633  -0.332   0.021   0.279   0.699
E W  1DVFA                 92    0.504 121.284  -1.608   0.018   0.088   0.893
E S  1DVFA                 93    0.493  57.803  -2.155   0.005   0.045   0.951
E T  1DVFA                 94    0.341  47.311  -1.107   0.018   0.088   0.893
E P  1DVFA                 95    0.415  58.818   0.021   0.020   0.205   0.775
B R  1DVFA                 96    0.236  53.975   0.372   0.021   0.451   0.528
B T  1DVFA                 97    0.137  19.044  -0.873   0.022   0.552   0.426
B F  1DVFA                 98    0.117  23.462  -0.777   0.004   0.514   0.481
B G  1DVFA                 99    0.118   9.318  -2.684   0.005   0.262   0.733
E G  1DVFA                100    0.458  36.045  -0.498   0.004   0.085   0.910
E G  1DVFA                101    0.328  25.821  -1.948   0.004   0.197   0.799
B T  1DVFA                102    0.155  21.457  -0.187   0.004   0.420   0.576
E K  1DVFA                103    0.398  81.930   1.603   0.001   0.900   0.099
B L  1DVFA                104    0.109  19.885  -0.018   0.001   0.959   0.040
E E  1DVFA                105    0.333  58.193   0.995   0.001   0.900   0.099
B I  1DVFA                106    0.133  24.568  -0.075   0.001   0.900   0.099
E K  1DVFA                107    0.695 142.961   1.391   0.004   0.197   0.799

Explain the output.  Go back.
```

---
